# The Role of the Orthopedist in the Spontaneous Foot Troubles Displayed by Soldiers on Active Service

**Published:** 1918

**Authors:** 


**The role of the Orthopedist in the Spontaneous Foot Troubles Displayed by Soldiers on active Service.** Professor FRÖHLICH, of the University of Nancy, read a paper describing the minor diseases of the foot and their treatment. He believed that except in rare instances of serious complications it was inadvisable to operate, as all such troubles were compatible with active service, or at least with the auxiliary services, and it was unnecessary to incur the long convalescence entailed by surgical treatment.

*Uncomplicated flat foot.* The ordinary army boot should be fitted with an entire sole made of cork and with the inside edge raised. The inner side of the heel should also be higher than the outer.

*Valgus, or weak ankle.* This is often due to a faulty synergy between the muscles of the leg or tibia and those of the fibula. It may be corrected by walking on the outside of the foot with the toes turned in.

*Arched foot.* For an excessively high instep a square-toed boot with raised toecap may be worn. The sole should be sufficiently hollowed to accommodate the back of the heel, but care must be taken to see that the anterior lip of the hollow does not fill in the whole of the vault of the foot. The thickened tissues of the sole must be treated by ablation in oblique layers without previous softening by liquids, and protection of the painful part by corn-plaster.

*Congenital club foot.* In men presenting themselves for military service this will have been already treated in infancy. When a slight deviation persists, a boot should be used which fixes the foot firmly in a slightly varus position: it should have a cork sole and the heel should be raised a little inside.

*Hallux Valgus* needs wide boots with accommodation inside for the protruding first metatarso-phalangeal articulation. A piece of cotton may be placed between the first and second toes to separate them and the bursa may be protected with a piece of corn-plaster.

*Congenital hallux varus and metatarsus varus.* The great toe should be held against the two neighbouring toes by means of a band of adhesive plaster, and wide boots should be worn with the foot turned slightly inward.

*Lateral deviations of the other toes or clino-dactylous conditions.* The toes should be fixed in correct position by adhesive plaster, or a boot provided with a dorsal pouch for the crooked toe may be worn.

*Hammer toes.* Square toed boots are necessary, large enough on the dorsal side not to rub against the folded and protruding great toe. The latter may be straightened and fixed to the two neighbouring toes by adhesive plaster, pieces of gauze being placed between them. The hard corns attendant on this deformity should be treated by ablation and protection by cornplaster.

*Metatarsalgia, Morton's disease, or break-down of the instep.* This may be due to inflammation and of short duration only, in which case rest and the wearing of a square-toed boot with a thick sole are all that is necessary. When there is a breakdown of the transversal plantar arch a strip of Velpeau, bandaged from the toes to the ankle, is effective.

*Supernumerary digits.* If these cause no real inconvenience it is sufficient to wear a boot with a pouch for the supernumerary digit in cases where it protrudes.

*Webbed toes* never cause trouble in walking and need no attention from the orthopedist.

*Localised apophysitis or ostitis of the small bones of the foot.* These are fairly frequent inflammatory lesions in young soldiers, but of short duration. Treatment necessitates a month's rest, mild revulsives and, when walking becomes possible, a boot with a special place for the painful bone.

*Subungual exostosis.* This may be tolerated with the protection of a hollowed corn-plaster and an appropriate boot preventing pressure on the exostosis. In such a case, however, it is better to operate, as a radical cure can be effected in 3 weeks.

*Onyxis or ingrowing toenail.* May be treated by the application of strands of thread, dipped in tincture of iodine, which raise the free edges of the nail.

*Corns* should be protected by fenestrated corn-plaster or treated by oblique ablation. Little wartlike appearances, which do not protrude but which may be very painful, should be destroyed by touching them at the centre with a drop of nitric acid every alternate day till cure.

*Excessive perspiratiou of the feet.* Preventive treatment consists in bathing the feet with a very weak dilution of formol and frequent change of socks. Socks with a ventilation hole in the anterior part of the heel may also be employed.

*Blisters.* To prevent these the boots must be flexible and kept well oiled; the feet also should be oiled before each long march.

*The subject of the second meeting of the session, May 18, at 10 : 00 A. M., was Aviation.*

